# Disintegration, Arrestation and Preventation of a Recurrence of Caries upon the Proximal Surfaces of the Teeth

**Published:** 1877-08

**Authors:** Marshall H. Webb

**Affiliations:** Lancaster, Pa.


### DISINTEGRATION, ARRESTATION AND PREVENTATION OF A RECURRENCE OF CARIES UPON THE PROXIMAL SURFACES OF THE TEETH.

BY MARSHALL H. WEBB, LANCASTER, PA.

(*Read before the Iowa Dental Society*, 1877.)

Disintegration of animal and vegetable tissue not only takes place, but it can also be observed throughout the whole universe of nature.

Castles built of the almost everlasting granite, and monuments of marble, erected to perpetuate the memory of departed worth, or to chronicle memorable events, as well as huge rocks in and by the sea, all gradually undergo disintegration through the play of the elements—air and water.

Although age by age, and century by century, may roll into eternity without there having been any visible manifestation of disintegration, yet, into the crevices already contained, or those being made in the mineral there will be deposited disintegrated vegetable tissue and granulated minerals; and as these crevices enlarge, and these particles accumulate, then, through moisture and the genial warmth of the sun, vegetable forms spring up from germinal matter finding lodgement within the fissures of the rock. Such growth continues, becomes more extensive, the fissures enlarge; and this taking place from century to century, castles and monuments crumble and chaos again ensues.

With regard to the animal kingdom, we find there are remote and proximate causes of disease and death. The remote causes of diseases and death are many—connected, directly and indirectly, with every element of the system; and can only be fully ascertained when pathology becomes an exact department of science. The proximate causes are few— consisting of the negation of fundamental principles of life.

Disintegration of dental tissue takes place through the action of acids. In uncleansed places upon enamel the *Leptoturix buccalis* accumulates; and since Loeber and Rottenstein announced that the parasite flourishes most luxuriantly in acid solution, it has been inferred that acids are thus more closely confined and hold to parts of tissue unwashed by saliva and uncleansed; and that the advance of decay is, therefore, quickened. The Leptothrix penetrates the clefts and interstices of disintegrated enamel, and, upon reaching the dentine, aids in accelerating the rapidity of caries.

Disintegration upon the proximate surfaces of the teeth commences, almost invariably, at or near the point or part of enamel in contact with the adjoining teeth—just above the point of contact in the upper, and just below it in the lower teeth—and after the dentine is reached, caries advance beneath the plate of enamel in all directions. Decay very rarely *commences* near the necks of the teeth, because they are free from contact at such part. The gum, when in a normal candition, encloses and protects these parts so perfectly that the cementum and the portion of enamel which may be beneath its margin (as well as gold when it has been properly inserted and its surface finely polished) is thoroughly protected from discoloration and disintegration.

When, through disintegration, the enamel of the proximate surfaces of the bicuspids and molars is penetrated, and the underlying dentine is attacked by caries, the cavity of decay should (excepting in rare instances) be opened into from the masticating surface, after separation by pressure of wedges of wood has been made, and the rubber dam applied. Even where but little structure *appears* to be disintegrated, this surface would be almost approached upon the perfect removal of the disintegration, and thus the plate of enamel would be liable to fracture, were hard surfaces to come in contact with it while food is undergoing comminution. It is far better to cut away this portion of enamel than to sacrifice such tissue along the entire proximate surface; besides, a better view can thus be facilitated, and the gold more perfectly introduced and solidly impacted. The adjoining sulcus or fissure is usually in such a condition as to require attention, and it should also be prepared and this operation completed at the time the other is performed. The entire cavity should be so prepared that no disintegrated enamel remains, and the edges be evenly and finely finished by means of fine "plug-finishing burs" and strips cut from emery cloth—from Nos. ½ and o—and a groove should also be cut around the cavity about where the enamel and dentine coalesce. This groove should be quite well marked along the buccal and palatal or lingual walls.

When a cavity has been prepared as just described, a *starting point* should be made in that portion of the cavity which extends near the neck of the tooth; not, however, in a direction where the plugs may, in any manner, be injured, but about where the dentine forms a coalesence with the other hard tissue of the part—the enamel or cementum. This pit should only be of sufficient depth to retain the small pieces of gold first introduced and impacted while other such pieces are being built upon them.

Gold in the form of cylinders and pellets should not be used in the performance of operations (unless cavities of decay are regarded as simple *holes*) because gold in such form cannot be carried to, and built in place (without the liability of movement when being introduced and impacted) so well as when light foil is folded upon spunk (which has been covered by kid) into a tape-like form of about six or eight lines in width, and then taken up with the foil-carriers and cut into parts about a line wide. A half leaf of foil for small, a whole leaf for medium, and two leaves for large operations, should be folded and cut in the manner just stated.

The fingers should never be brought in contact with gold, because this, as well as moisture of any kind, will bring about irreparable injury so far as that portion of gold is concerned. The law of affinity between particles must be obeyed—cohesion must not be impaired—else an enduring, useful, and beautiful structure cannot be erected; neither can the precious atoms, strong as they are "in solid singleness," exhibit enduring strength unless the gold be closely packed and the combination of the atoms be dense. Pure; and therefore, cohesive gold should be used for all operations which are intended to *best* subserve the purpose of disintegrated or lost tissue, but the highest type of operations is not attained by the use of cylinders and pellets, because, in additions to reasons already given, cohesive gold can not be used so as to bring about a result as nearly perfect as when foil is prepared as before described, and carefully and thoroughly introduced and impacted, and properly formed and finely finished.

The gold should not only be built against every part of dental tissue, but be impacted as perfectly as possible along and over the margins of enamel, and fully out to the proximate surface of the teeth adjoining—using a part of such surface as a matrix. In such cases, as throughout almost all operations, all the gold should be impacted by aid of the mallet. The electro-magnetic mallet, *when properly adjusted, understood and operated,* is far superior to any other such instrument. By its aid an operation can be more easily, rapidly, and perfectly performed than in any other manner. For this priceless boon we are indebted to Dr. W. G. A. Bonwill, of Philadelphia, who, if he did not first conceive of the idea of the the application of electrity to this purpose, first produced a practical electro-magnetic mallet. Modifications of the instrument have been made by others, but such of these changes as are worth adoption add to, rather than detract from, the value of the electro-magnetic mallet, and increase rather than cancel, the debt of gratitude we all owe to Dr. Bonwill.

After the gold has been impacted, piece by piece, so the substitution of lost tissue is complete, a fine "separating file" should be used to cut away the surplus material, and to aid in making the filling conform to the original contour of the teeth; after which strips cut from emery-cloth of the finest grades, should be so manipulated as to more perfectly finish the surface of the gold and to leave the parts nicely formed. All this must be done previous to the removal of the rubber dam, after which the finishing should be completed by the use of finely pulverized pumice and silex, both mounted upon linen tape. These may be followed by fine rouge applied upon a strip of fine chamois skin. This leaves a finer finish than a burnisher, but not so bright a polish. The finishing of the gold upon the masticating surface should be done with corrundum cones and Hindoostan stones, followed by pumice mounted upon wood, leather or rubber points. A sharp, fine, "plug-finishing burr" may be required to aid in making the filling conform to the depressions in the masticating surface. All these cones, stones, points and burrs should be operated with the "dental engine."

Operations upon proximate surfaces of the cuspid and incisor teeth should be performed in a manner somewhat similar to that already indicated, as well as in accordance with correct and efficient judgment. Gold should not be exposed to view whenever it is possible to prevent it, but where exposure is unavoidable, and the operation perfectly performed, and the filling artistically formed and its surface finely and beautifully finished, the gold will not be repulsive to the eye.

One should always endeaver to perform such operation, and so finish each filling that it shall conform to the line which defined the original figure. This contour line should be restored to that degree commensurate with the extent of destruction of tissue. The contour of the parts operated upon should be so restored in gold that the prominent portion of the solidly impacted, well anchored, and finely finished gold comes in contact with the adjoining tooth, (or with a filling in such tooth) near the buccal and masticating surfaces of the teeth, and in such a manner as to leave the margins of enamel free from contact with another tooth at every point.

When such operations as referred to have been properly performed and a space, as in nature, remains at and near the necks of the teeth, and gold comes in contact with gold, or the normal structure of the tooth adjoining, near the buccal and masticating surfaces, and there be freeness of contact of enamel, caries will rarely if ever recur; because (in addition to the operation having been quite perfectly performed, and in accordance with the correct principle) the margins of enamel against which the gold has been built and finely and evenly finished, are almost constantly quite well washed by the saliva which is kept in motion by the action muscles of the cheek, lips, and tongue.

It has been asserted that gold is "a purely mechanical remedy" for the prevention of a reccurrence of decay, while the oxide formed from silver or tin, or both, as in an amalgam, "turns the teeth to a dark color and checks caries." This "preservation" (?) has been said to be "chemical."

If carious dentine has been properly and thoroughly removed, and every particle of disintegrated enamel taken away from and about the margin of the cavity, and gold be properly prepared, introduced, and impacted against every part of the prepared structures without there having been the slightest movement of such gold, and the fillings have then been properly finished down to, and polished with, the surrounding enamel, there will then be no space for moisture to enter, and there can therefore be no chemical action and no electric currents evolved between gold and calcium. The only manner in which this can then occur is by disintegration at some point or points about or around the margin of enamel, against which the gold has been impacted, and this must have been imperfectly done, or the margins of enamel must not have been free, else this disintegration would not, in all probability, have taken place. If the operation be properly performed, and the margin of enamel be free from contact with the teeth adjoining, and have been so shaped as to be cleansed by the action of saliva, the parts cannot well be otherwise than free from a recurrence of caries.

It is a difficult task to thus perfectly perform operations, and especially in some instances and under some conditions. To accomplish all which I have just indicated—that almost any of the duties of the faithful and conscientious practitioner may be performed—it is necessary for one to understand the anatomical, physiological, and pathological conditions and relations; in a word, diseased tissues cannot be properly treated—really first class operation performed—without some such knowledge as well as keen perceptions and the exercise of correct and efficient judgment.

Almost the same care is required for the preservation of the teeth by the arrestation of caries, and the prevention of its recurrence, through the filling of the cavity of decay with another material, such as tin foil, as with gold, notwithstanding the assertion that an amalgamated mass *chemically preserves* the remaining structure. Oxidation cannot take place where moisture cannot reach; hence, where moisture is there may not only be such oxidation but also chemical action, evolution of electric currents, and disintegration and destruction of tissue, except in those rare instances where there is *black* decay—where the salts formed by decomposition of the mineral portion of the teeth, are insoluable and become paralyzed in a negative position to the decomposition agent.

It seems fair to conclude that, in consideration of all this, there is no point about any of the materials which have so far been used for the preservation of the teeth that is equal to gold even if, as has been asserted, it is "a purely mechanical remedy," in contradistinction to amalgam with its "*chemically preservative influence.*" If a fully qualified practitioner and capable operator desires to perform operations and to preserve the teeth as perfectly as possibly, he should (unless it be in very rare instances) make use of gold as a permanent filling for the preservation of the valuable organs, he may be called upon to operate, and if he does not possess the ability to perform operations as they ought to be performed, then, rather than attempt to save these organs by unskillfully and imperfectly inserting gold in cavaties of decay, one should make use of some material which, because of its plasticity, may enable him to more perfectly perform the operation.

Failure in dental operations is attributable, in the majority of instances, to the very imperfect manner in which such operations are performed, and all this should be duly and and honestly considered and acknowledged before the cause of failure is attributed to acids and electric currents evolved from chemical action.—*Missouri Dental Journal.*

